# Supplementary material for: The Small RNA Universe of Capitella teleta
Source: Front Mol Biosci. 2022 Feb 25;9:802814. doi: 10.3389/fmolb.2022.802814 (PMC8915122; doi:10.3389/fmolb.2022.802814)
Supplement: Supplementary file 1 [file DataSheet1.ZIP › Supplement/known/CAPTEscaffold_453_19005.pdf]

Provisional ID	:	CAPTEscaffold_453_19005
Score total	:	4514.2
Score for star read(s)	:	3.9
Score for read counts	:	4503.9
Score for mfe	:	1.8
Score for randfold	:	1.6
Score for cons. seed	:	3
Total read count	:	8846
Mature read count	:	8780
Loop read count	:	0
Star read count	:	66

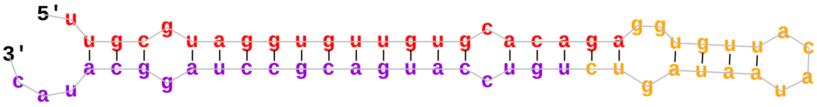

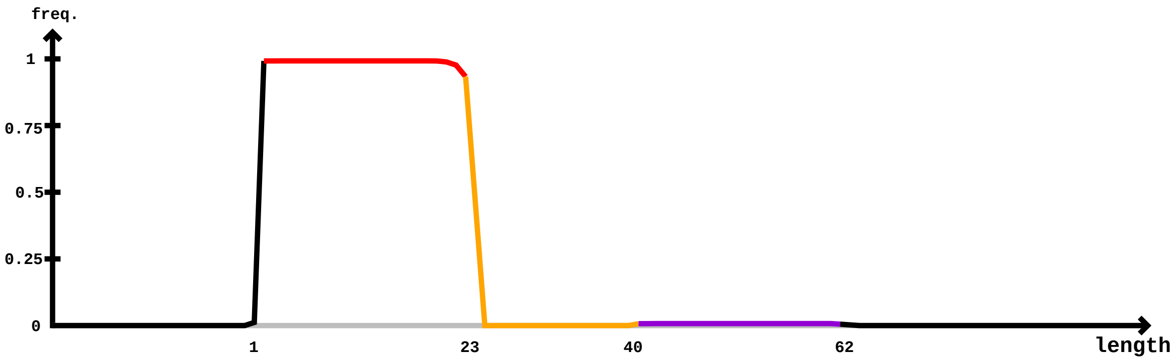

Mature **Star**

```
5'- uccaaucguuaaccauagauuugcguagguguugugcacagagguguuacauaauagucuguccaugacgccuaggcauacccgugguugacgcaucuucaaauuaacaaag  -3'  obs
    uccaaucguuaaccauagauuugcguagguguugugcacagagguguuacauaauagucuguccaugacgccuaggcauacccgugguugacgcaucuucaaauuaacaaag       exp
    uccaaucguuaaccauagauuugcguagguguugugcacagagguguuacauaauagucuguccaugacgccuaggcauacccgugguugacgcaucuucaaauuaacaaag       known
    ......((((((((((.(...(((.(((((((((((.(((((..((((....)))).))))).))))))))))).)))...).))))))))))...................  reads  mm  sample

    ...................uuugcguagguguugugcac.........................................................................  3     0   seq
    ...................uuugcguagguguugugcaca........................................................................  20    0   seq
    ...................Auugcguagguguugugcacag.......................................................................  2     1   seq
    ...................uuugcguagguguugugcacag.......................................................................  44    0   seq
    ...................uuugcguagguguugugcacagU......................................................................  2     1   seq
    ...................Cuugcguagguguugugcacaga......................................................................  4     1   seq
    ...................uuugcguagguguugugcacaga......................................................................  28    0   seq
    ....................uugcguagguguugugca..........................................................................  3     0   seq
    ....................uugcguagguguugugcac.........................................................................  29    0   seq
    ....................uugcguagguguugugcaU.........................................................................  1     1   seq
    ....................uugcguagguguugugcaca........................................................................  80    0   seq
    ....................Augcguagguguugugcaca........................................................................  1     1   seq
    ....................uugcguaggAguugugcaca........................................................................  1     1   seq
    ....................uugcguagguguugugcacag.......................................................................  334   0   seq
    ....................uugcAuagguguugugcacag.......................................................................  1     1   seq
    ....................uugcguagAuguugugcacag.......................................................................  1     1   seq
    ....................uugcguagguguuguAcacag.......................................................................  1     1   seq
    ....................Augcguagguguugugcacag.......................................................................  1     1   seq
    ....................uugcguagguguugugcacaAa......................................................................  1     1   seq
    ....................uugcguGgguguugugcacaga......................................................................  1     1   seq
    ....................Augcguagguguugugcacaga......................................................................  26    1   seq
    ....................uugcguagguguugugcacaga......................................................................  8098  0   seq
    ....................uugcguagguguugugcacUga......................................................................  1     1   seq
    ....................uuAcguagguguugugcacaga......................................................................  2     1   seq
    ....................uugcguagguguAgugcacaga......................................................................  3     1   seq
    ....................uugcguaAguguugugcacaga......................................................................  1     1   seq
    ....................uugcguagguguugugcacaCa......................................................................  2     1   seq
    ....................uCgcguagguguugugcacaga......................................................................  2     1   seq
    ....................uugcguagguguuguUcacaga......................................................................  1     1   seq
    ....................uugcguagguguugugcaUaga......................................................................  2     1   seq
    ....................uugcguagguguugugcaGaga......................................................................  1     1   seq
```

```
                    Mature                                      Star
uccaaucguuaaccauagauuugcguagguguugugcacagagguguuacauaaauagucuguccaugacgccuaggcauacccgugguugacgcaucuucaaauuaacaaag
....................uugcguagguguuguCcacaga.......................................................................   1    1    seq
....................uugcguagguguugugcGcaga.......................................................................   1    1    seq
....................uugAguagguguugugcacaga.......................................................................   3    1    seq
....................uugcguagguUuugugcacaga.......................................................................   1    1    seq
....................uugcguagguguugugcaAaga.......................................................................   1    1    seq
....................uugcguagguguugAgcacaga.......................................................................   2    1    seq
....................uugcguaggugCugugcacaga.......................................................................   4    1    seq
....................uugcgCagguguugugcacaga.......................................................................   1    1    seq
....................Gugcguagguguugugcacaga.......................................................................   9    1    seq
....................uugcguagguAuugugcacaga.......................................................................   1    1    seq
....................uugcguagguguugugGacaga.......................................................................   2    1    seq
....................uugcguagCuguugugcacaga.......................................................................   1    1    seq
....................uugcguagguguugugcacGga.......................................................................   1    1    seq
....................uugcguagguguugugcacagG.......................................................................   9    1    seq
....................uugcgAagguguugugcacaga.......................................................................   2    1    seq
....................Cugcguagguguugugcacaga.......................................................................   1    1    seq
....................uugcAuagguguugugcacaga.......................................................................   2    1    seq
....................uugcguagguguCgugcacaga.......................................................................   1    1    seq
....................uugcguaggugAugugcacaga.......................................................................   1    1    seq
....................uugcguagguguuAugcacaga.......................................................................   2    1    seq
....................uAgcguagguguugugcacaga.......................................................................   2    1    seq
....................uugUguagguguugugcacaga.......................................................................   2    1    seq
....................uugcguaggAguugugcacaga.......................................................................   6    1    seq
....................uuCcguagguguugugcacaga.......................................................................   1    1    seq
....................uugGguagguguugugcacaga.......................................................................   1    1    seq
....................uugcguagguguugugcacagU.......................................................................   13   1    seq
....................uugcguaCguguugugcacaga.......................................................................   1    1    seq
....................uugcguagguguugugcacagaA......................................................................   7    1    seq
....................uugcguagguguugugcacagaU......................................................................   2    1    seq
......................gcguagguguugugcacag........................................................................   1    0    seq
......................gcguagguguugugcacaga.......................................................................   1    0    seq
............................................................uguccaugacgccuaggcaua................................   20   0    seq
............................................................ugAccaugacgccuaggcauac...............................   1    1    seq
............................................................uguccaugacgccuaggcauac...............................   42   0    seq
.............................................................guccaugacgccuaggcauacc..............................   1    0    seq
..............................................................uccaugacgccuaggcauacc..............................   2    0    seq
```
